# *In vitro* evaluation of the anti-microbial efficacy of natural root canal irrigants activated with gamma radiation

**DOI:** 10.6026/973206300211471

**Published:** 2025-06-30

**Authors:** Shrishtee Priya, Harleen Kaur Sohi, Subasish Behera, Priyanka Pandey, Bibyaswan Chakrabarti, Soumyaranjan Nanda, Miral Mehta

**Affiliations:** 1 Department of Conservative Dentistry and Endodontics, Vananchal Dental College, Garhwa, Jharkhand, India; 2 General Dentist in Massachusetts, 416 Tyler Street, Pittsfield, 01201 Massachusetts, United States; 3 Department of Conservative Dentistry and Endodontics, Government Dental College and Hospital (SCB), Manglabag, Cuttack, Odisha, India; 4 Department of Conservative Dentistry and Endodontics, Bhabha College of Dental Sciences, Bhopal, Madhya Pradesh, India; 5 Department of Pediatric & Preventive Dentistry, College of Dental Science and Hospital, Rau, Indore, Madhya Pradesh, India; 6 Department of Endodontics, Private Practitioner, Cuttack, Odisha, India; 7 Department of Pediatric and Preventive Dentistry, Karnavati School of Dentistry, Karnavati University, Gandhinagar, Gujarat, India

**Keywords:** Root canal irrigants, natural extracts, gamma radiation, antimicrobial efficacy, *Enterococcus faecalis*, *In vitro* study

## Abstract

Neem and Tulsi were investigated as natural root canal irrigants with and without gamma radiation activation to evaluate their performance against *Enterococcus faecalis*. In total, 60 affected teeth were divided into six groups and each group was treated with a different irrigant. Neem that received gamma-irradiation had the top reduction of bacteria (96.5%), followed by Tulsi and sodium hypochlorite. The effectiveness of non-irradiated extracts was moderate, compared to the mild results found from saline. Gamma radiation made herbal irrigants much more effective, showing their potential for use as biocompatible treatments.

## Background:

The success of endodontic therapy is largely due to properly disinfecting the root canal system. Microbial eradication cannot be accomplished just by using mechanical methods because the fin, isthmus, accessory canal and apical delta shapes inside the root canals make it difficult [1]. *Enterococcus faecalis* is singled out among the microbes in endodontic infections because it can penetrate the dentin, withstand the tough conditions found inside, build up biofilms and resist commonly used canal medications. Because this pathogen is often connected to post-treatment disease and inflamed roots, strong antibiotics should be used in root canal therapy [2]. Sodium hypochlorite (NaOCl) has for years been recognized as the leading choice for root canal irrigation, mostly due to its strong ability to fight bacteria and viruses, break down tissue and cost less than other chemicals. Chlorhexidine (CHX), a cationic bisbiguanide, is liked for sticking to the hair and skin, as well as for killing bacteria of different types [3]. Both NaOCl and CHX can cause issues, even though they are used a lot. If this drug is placed outside the root's tip, it may be toxic to cells and can result in major inflammatory damage in the area. Chlorhexidine is unable to dissolve tissues and may sometimes result in staining or allergies in people with sensitive skin [2, 3, 4- 5]. Because of these issues, experts have looked into safer, more compatible methods that still work against microbes. Lately, plant-based therapies have seen more attention in the field of endodontic research. More people are becoming aware that natural extracts have multiple pharmacological effects, such as fighting microbes, soothing inflammation, working as antioxidants and helping wounds heal [4]. Native to the Indian subcontinent, neem (*Azadirachta indica*) has bioactive compounds that powerfully fight both bacteria and fungi in the body. Tulsi (*Ocimum sanctum*) is another herb used for its health benefits, which come from having a lot of eugenol and ursolic acid compounds. Scientists have found that both plants can treat oral bacteria found in endodontic infections. Although these extracts are promising, antibacterial activity is typically believed to be lower than that of usual antibiotic drugs when looked at separately. Some scientists have searched for new ways to better use these herbal medicines. Experts also use gamma radiation, which delivers high-energy ionizing rays able to induce changes in the structure of organic compounds [6]. Gamma rays often reach the center of materials and damage them by creating many free radicals and reactive oxygen species (ROS). Chemical treatments often boost how easily plant extracts can work and how well they prevent diseases, while still preserving their character and safety. Gamma irradiation is able to sterilize raw herbal preparations and remove harmful microbes from them [7]. Even though gamma irradiation is thought to work well, researchers have not fully studied its use to improve the effectiveness of root canal irrigants from plant sources. Since there is a worldwide movement toward using dental materials that are kinder to the environment and healthier for patients, it is now important to provide scientific evidence for these integrative methods. Therefore, it is of interest to compare the antimicrobial effects of Neem and Tulsi extracts, both before and after gamma radiation activation.

## Materials and Methods:

## Study design and sample preparation:

An *In vitro* experimental study was conducted using 60 freshly extracted, single-rooted human mandibular premolars with straight canals and fully formed apices. Teeth with fractures, resorption, or calcifications were excluded. The specimens were cleaned of soft tissue debris and stored in 0.1% thymol solution until use.

## Access cavity and canal preparation:

Access cavities were prepared using a high-speed diamond bur under water spray. Working length was determined by inserting#10 K-file into the canal until visible at the apex and subtracting 1 mm. Root canals were instrumented using ProTaper rotary files up to size F3, irrigated with saline during the procedure to avoid chemical interaction with test irrigants. After instrumentation, all canals were flushed with 5 mL of distilled water and dried using sterile paper points.

## Grouping and irrigant preparation:

The samples were randomly divided into six experimental groups (n = 10 per group):

[1] Group I: Normal saline (negative control)

[2] Group II: Neem extract (unirradiated)

[3] Group III: Tulsi extract (unirradiated)

[4] Group IV: Neem extract exposed to gamma radiation (5 kGy)

[5] Group V: Tulsi extract exposed to gamma radiation (5 kGy)

[6] Group VI: Sodium hypochlorite (2.5%) (positive control)

## Preparation of plant extracts:

Neem leaves and Tulsi leaves were shade-dried and ground into a fine powder. Each was subjected to Soxhlet extraction using ethanol as a solvent. The extracts were filtered and evaporated to dryness using a rotary evaporator. Final concentrations were adjusted to 10% (w/v) using sterile distilled water. For Groups IV and V, the extracts were exposed to 5 kGy gamma radiation using a Cobalt-60 source at a certified irradiation facility.

## Bacterial contamination:

Standard strains of *Enterococcus faecalis* (ATCC 29212) were rehydrated and cultured in brain-heart infusion (BHI) broth. Each canal was inoculated with 10 µL of bacterial suspension (~1.5 x 10^8^ CFU/mL) and incubated at 37°C for 21 days to allow for biofilm formation. Fresh broth was replenished every 48 hours under aseptic conditions.

## Irrigation protocol:

Following incubation, canals were irrigated with 5 mL of the respective test irrigants using a 30-gauge side-vented needle over a period of 1 minute. The needle was positioned 1 mm short of the working length and moved gently in an up-and-down motion during irrigation. Canals were then dried with sterile paper points.

## Microbial sampling and CFU Count:

Sterile absorbent paper points were introduced into each canal for 60 seconds to absorb residual bacteria. The paper points were transferred into tubes containing 1 mL sterile saline, vortexed for 30 seconds and serially diluted. Aliquots were plated on BHI agar and incubated at 37°C for 48 hours. Colony-forming units (CFUs) were counted manually using a digital colony counter.

## Statistical analysis:

Data were tabulated and analyzed using SPSS version 25.0 (IBM Corp., Armonk, NY). One-way analysis of variance (ANOVA) was used to compare mean CFU counts among the groups, followed by Tukey's post hoc test. A p-value of less than 0.05 was considered statistically significant.

## Results:

The antimicrobial activity of each irrigant was evaluated by measuring the mean colony-forming units (CFUs) of *Enterococcus faecalis* remaining after irrigation. A statistically significant difference was observed among the six groups (p < 0.001), as shown in Table 1 (see PDF). Group IV (gamma-irradiated Neem) demonstrated the lowest mean CFU count (3.3 x 10^3^ ± 0.9), showing a 96.5%reduction in bacterial load, followed closely by Group V (gamma-irradiated Tulsi) with a 93.8% reduction. Group VI (sodium hypochlorite) exhibited a 91.2% reduction, validating its known efficacy. Groups II and III, which used unirradiated Neem and Tulsi, respectively showsed moderate antimicrobial effects with reductions of 65.4% and 60.1% respectively. As expected, Group I (normal saline) showed negligible antimicrobial activity (Table 1 - see PDF). Statistical analysis using ANOVA revealed significant differences among the groups (F = 115.2, p < 0.001) and Tukey's post hoc test confirmed that the gamma-irradiated extracts (Groups IV and V) had significantly greater antimicrobial efficacy than their non-irradiated counterparts (p < 0.05).

## Discussion:

The study tested whether Neem (*Azadirachta indica*) and Tulsi (*Ocimum sanctum*) irrigants are effective against microbes, comparing their effects when used in native form or after being activated by gamma radiation. In the study, irradiated extracts of Neem and Tulsi performed better at fighting *Enterococcus faecalis* than non-irradiated samples and were as effective as sodium hypochlorite. Radiologically enhanced herbal methods may have a positive role in endodontic disinfection procedures. Because *E. faecalis* withstands severe pressure, sticks to surfaces and penetrates the dentin, it is a major cause of persistent root canal infections [1]. Many written reports prove that this bacterium can resist modern disinfectants for endodontics, which is why scientists are now searching for alternative biocompatible treatments [2, 3]. Recently, scientists have noticed that plant-based extracts can widely affect harmful effects and are unlikely to produce significant side effects [4]. A lot of research has been done on neem for its abilities to fight bacteria, fungi and viruses and control inflammation. Many think that the antimicrobial effects of neem are due to nimbidin, azadirachtin and quercetin [5, 6]. Tulsi, like the other herbs, includes eugenol, ursolic acid and carvacrol, which disrupt the membranes of microbes and stop them from reproducing [7, 8]. We found that Neem and Tulsi extracts had moderate antimicrobial effects against *E. faecalis*, which agrees with other studies that found a similar result using them in endodontic treatment [9, 10]. The antimicrobial effect of these herbal extracts increased when they were exposed to gamma radiation at 5 kGy. Ionization due to gamma radiation on phytochemicals can make them form free radicals and reactive oxygen species, which in turn may increase the bacteria-fighting power of the original material [11, 12]. We saw this difference clearly, as the CFU counts in the Neem and Tulsi groups were reduced by 96.5% and 93.8% after irradiation. It has similarly been found that gamma radiation can sterilize and stimulate parts of herbal compounds when applied in food and pharmaceutical fields [13].

Scientific studies prove that exposing herbal extracts to gamma radiation produces extracts with greater antioxidant and antibacterial power, as the complex molecules become broken down into simpler active compounds [14]. We found much the same results as Ahmed and Hassan (2023) did when they found that adding gamma radiation to fennel seed extract improves its antibacterial qualities while not affecting its safety [15]. Among the possible solutions, sodium hypochlorite is chosen as the standard because it dissolves tissue and kills bacteria too well. Yet, because this substance can damage the tissues at the tips of roots and easily cause irritation or allergies, it is sometimes not suitable for everyone [16, 17-18]. Alternative herbal remedies, especially if they have been improved with either radiological or chemical steps, provide a safer process for cleaning out tooth canals. An exciting factor in this study is that the gamma-irradiated Neem worked as well as sodium hypochlorite, indicating that irradiated phytochemicals may be as good as or better than conventional treatments in fighting bacteria without causing the same harmful effects. Yet, understanding exactly how gamma radiation allows plant extracts to kill microbes at a molecular level is still needed. Nevertheless, there are some weaknesses in this study. The testing was done under controlled conditions in a lab, which is not the same as what you find in a real root canal. Only *E. faecalis* was used as the test organism in this study. Further work should consider multispecies biofilms as well as check the herbal extracts' ability to be toxic or penetrate teeth after radiation in animals.

## Conclusion:

The combined use of gamma radiation and herbal irrigants appears to improve the disinfection of root canals. This method can change the way endodontic therapy works by merging strong antibacterial action with the need for safe treatments.
